# “Salud Mamaria”, an internet-based psychoeducational program during the breast cancer diagnosis process: Protocol for a randomized controlled trial

**DOI:** 10.1016/j.conctc.2024.101397

**Published:** 2024-11-26

**Authors:** Reyna Jazmín Martínez-Arriaga, Alejandro Dominguez-Rodriguez, Sergio Osvaldo Meza-Chavolla, Yineth Alejandra Muñoz-Anacona, Adrián Antonio Cisneros-Hernández, Joel Omar González-Cantero, Leivy Patricia González-Ramírez, Paulina Erika Herdoiza-Arroyo, Norma Alicia Ruvalcaba-Romero, Fabiola Macías-Espinoza, Said Jiménez

**Affiliations:** a Departamento de Clínicas de Salud Mental, Centro Universitario de Ciencias de la Salud, Universidad de Guadalajara, 950 Sierra Mojada St, Independencia, 44340, Guadalajara, Jalisco, Mexico; b Department of Psychology, Health and Technology, University of Twente, Drienerlolaan 5, 7522, NB, Enschede, Netherlands; c Unidad de Detección y Diagnóstico Cáncer de Mama, Instituto Mexicano del Seguro Social, OOAD Jalisco. Belisario Domínguez 3005 Av., Jardines de Santa Isabel, 44300, Guadalajara, Jalisco, Mexico; d Departamento de Ciencias del Comportamiento, Centro Universitario de los Valles, Universidad de Guadalajara, 46600, Carretera Guadalajara – Ameca Km. 45.5, Ameca, Jalisco, Mexico; e Departamento de Proyectos de Comunicación, Centro Universitario de Arte, Arquitectura y Diseño, Universidad de Guadalajara, 5075 Independencia Norte, Huentitán El Bajo, 44250, Guadalajara, Jalisco, Mexico; f School of Medicine and Health Sciences, Tecnologico de Monterrey, Guadalajara Campus. 2514 Gral Ramón Corona Av., Nuevo México, 45201, Zapopan, Jalisco, Mexico; g Faculty of Medical, Health and Life Sciences, Universidad Internacional del Ecuador UIDE, Jorge Fernandez, 170411, Quito, Ecuador; h Departamento de Psicología Básica, Centro Universitario de Ciencias de la Salud, Universidad de Guadalajara, 950 Sierra Mojada St., Independencia, 44340, Guadalajara, Jalisco, Mexico; i Departamento de Psicología Aplicada, Centro Universitario de Ciencias de la Salud, Universidad de Guadalajara, 950 Sierra Mojada St., Independencia, 44340, Guadalajara, Jalisco, Mexico; j School of Medicine and Health Sciences, Tecnologico de Monterrey. Canal de Miramontes, Coapa, San Bartolo el Chico, Tlalpan, 14380, Mexico City, Mexico

**Keywords:** Internet-Based psychoeducational program, Breast health, Breast cancer, Randomized controlled trial, Cancer screening, Cancer diagnosis

## Abstract

**Background:**

Some of the key challenges during the breast cancer diagnosis process include a lack of information and negative psychological consequences, such as distress and anxiety about the process. Implementing a psychoeducational program during the diagnosis process may enhance the well-being of women. “*Salud Mamaria*” is an Internet-Based Psychoeducational Program (IBPP) that comprises three interventions: A (“Improving Your Health Habits and Self-Care”), B (“Waiting for the Result of Your Biopsy”), and C (“Supporting You After Your Breast Cancer Diagnosis”).

**Objective:**

1) To evaluate changes in the study variables following each of the three interventions (A, B, and C), and 2) To assess the differences in study variables between the IBPP and an active control group.

**Methods:**

This is a superiority trial employing an experimental design with two independent groups: an experimental group and an active control group. All participants will be randomized to one of the two conditions. Anxiety symptoms, negative screening of consequences, sense of coherence, satisfaction with the intervention, and system usability will be measured. Patients will be assigned to an intervention based on their clinical situation: without cancer suspicion (A), with cancer suspicion (B), or diagnosed with cancer (C). Questionnaires will be administered via the online platform before and after each intervention.

**Conclusions:**

A psychoeducational program implemented during the breast cancer screening and diagnosis process may promote the health and well-being of women. It may also encourage adherence to medical screening recommendations, mitigating the lack of information and reducing associated distress.

**Trial registration:**

ClinicalTrials.gov NCT05830461.

## Highlights

• “Salud Mamaria” provides evidence-based cancer information through a digital tool.
• This digital tool can be expanded to Mexico and other Spanish-speaking countries.
• “Salud Mamaria” was created by specialists from different disciplines.
• The tool was designed based on the user's characteristics, to reduce the drop-out.
• The program consists of 3 interventions, to reach women in different circumstances.

## 1 Introduction

According to the World Health Organization, its guide to cancer early diagnosis emphasizes that early detection and screening are two critical strategies in the process, as they facilitate the identification of symptoms during the early stages of the disease. Additionally, they enable the detection of asymptomatic cancer cases in otherwise healthy populations [1]. In certain populations, approximately 50 % of women are aware of the cancer screening process; however, the proportion of women with knowledge about breast cancer risk factors is significantly lower [2]. Moreover, knowledge about breast cancer and preventive methods is influenced by factors such as education level, income, and type of employment [3]. While health information is crucial, it alone does not necessarily result in significant behavioral changes, particularly in the context of breast cancer screening [4]. Therefore, it is essential to consider additional factors, such as psychological variables, that can influence the breast cancer diagnostic process.

Although there are limited studies on psychological conditions during cancer screening, this process can cause psychological discomfort due to the possibility of a positive cancer diagnosis, as cancer is still socially associated with death [5]. Additionally, other challenges may arise, such as existential questioning, deterioration of self-concept, financial concerns, and treatment-related difficulties [6]. While lower levels of psychological distress have been reported during the pre-diagnosis stage compared to subsequent stages, Hispanic and uninsured women have been shown to experience higher levels of distress during breast cancer screening [7]. Specifically, some psychological consequences observed in individuals diagnosed with cancer include denial, depressive and anxiety symptoms, and/or anger [8], along with a decline in psychological well-being [9]. Furthermore, issues such as self-concept deterioration, body image disturbances, sexual problems, and difficulties in social relationships have also been reported [6]. As a result, developing psychosocial care alternatives for individuals undergoing these diagnostic processes becomes highly relevant.

Online interventions present a viable alternative to face-to-face interventions, as they address challenges such as geographical distance and scheduling constraints [10]. These interventions are typically accessed via smartphones, tablets, and other mobile devices through applications, websites, and social networks. Some of these interventions focus on providing psychoeducational information about breast cancer [[11], [12], [13]]. Benefits of online interventions include reducing fatigue and depression in individuals with cancer [14], as well as improving quality of life and dietary habits in female cancer survivors [15]. However, despite their clinical potential in enhancing the health of breast cancer patients, future research with more rigorous experimental designs is necessary [16]. Age, gender, and cancer type have been identified as mediators of online intervention acceptance. While younger individuals tend to show higher acceptance rates, older adults are more likely to adhere to online interventions aimed at reducing cancer-related distress once they are enrolled [17]. Therefore, identifying and addressing barriers that prevent older adults from initially accepting online interventions is crucial. This underscores the importance of increasing the number of interventions targeting the adult population.

Furthermore, evidence-based psychology emphasizes the importance of considering environmental and population conditions (e.g., age, gender, and racial group) when designing psychological interventions [18]. Such adaptations may help reduce dropout rates, a significant challenge in the design and implementation of online interventions [19].

“*Salud Mamaria*” (Breast Health, in English) is an Internet-Based Psychoeducational Program (IBPP) designed for women undergoing breast cancer screening. The IBPP was developed based on findings from a previous study that analyzed women's knowledge of diagnostic tests for breast cancer and the psychological discomfort experienced during screening [20]. The study revealed that the average age of participants was 53 years, underscoring the importance of creating a digital tool that is adult-friendly. Additionally, 38 % of participants were unaware of the diagnostic tests they were about to undergo, and most (84 %) attended the hospital without receiving complementary information [20]. In terms of psychological discomfort, 50 % of participants reported anxiety symptoms, while 34 % experienced depressive symptoms. These findings highlight the need to address the psychological discomfort and negative psychological consequences associated with the screening process, while also incorporating positive psychological variables, such as the sense of coherence. Sense of coherence has been shown to predict higher levels of quality of life in early-stage breast cancer patients at 3- and 5-year follow-ups [21]. This concept refers to an individual's general life orientation, which guides them in identifying and utilizing resources necessary to maintain health, particularly during stressful periods [22]. A strong sense of coherence has also been associated with better health outcomes in adult women [23].

### 1.1 Objectives

The objectives of this study are:

1. To evaluate the changes in the study variables (anxiety, negative psychological consequences, and sense of coherence) following each of the three interventions: A (“Improving Your Health Habits and Self-Care”), B (“Waiting for the Result of Your Biopsy”), and C (“Supporting You After Your Breast Cancer Diagnosis”) of the IBPP.

Hypothesis: The IBPP will reduce symptoms of anxiety and negative psychological consequences while increasing the levels of sense of coherence in participants.

2. To evaluate the differences in the study variables between the IBPP and the active control group.

Hypothesis: Women who participate in the IBPP will experience a greater reduction in symptoms of anxiety and negative psychological consequences, as well as a greater increase in sense of coherence, compared to the active control group.

## 2 Methods

### 2.1 Study design

**Fig. 1:**
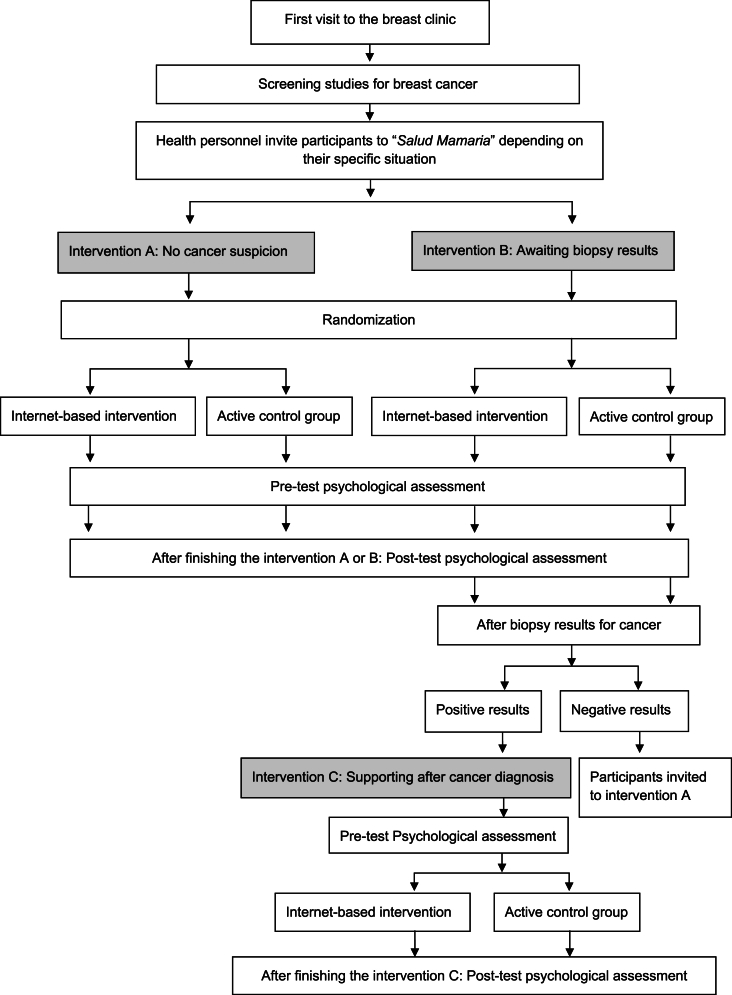
Study procedure and data collection.
b) The active control group will receive visual material covering the same topics as those presented in the videos on the virtual platform. This material will include educational information on the topics covered in each module of interventions A, B, and C. An expert psychologist will conduct assessments before and after each intervention. In accordance with ethical principles, participants in the active control group will be invited to access the videos on the platform after the evaluations.

This is a superiority trial [24] with an experimental design involving two independent groups: the experimental group (IBPP) and the active control group (visual material):

a) The experimental group will participate in the online psychological program delivered via a virtual platform. The IBPP consists of three interventions (A, B, and C), each containing self-administered modules tailored to different patient groups: 1) those without a suspicion of cancer (intervention A), 2) those with a suspicion of cancer awaiting biopsy results (intervention B) and 3) those who receive a positive cancer result (intervention C). Participants in each intervention (A, B, and C) will undergo assessments before (pre-measurement) and after (post-measurement) receiving the program content. The participant flow chart is presented in Fig. 1.

Furthermore, this protocol adheres to the Standard Protocol Items: Recommendations for Interventional Trials (SPIRIT) statement [25] (Appendix A).

### 2.2 Randomization and allocation

All participants attending the Breast Cancer Detection and Diagnosis Unit (Breast Clinic) for screening and/or diagnostic evaluation will be invited to participate in the program.

A stratified randomization will be applied. Participants will be stratified based on their clinical status: no suspicion of cancer (intervention A) or awaiting biopsy results (intervention B). Participants will be randomly assigned to either the intervention (A or B) or the control arm within each stratum. For those awaiting biopsy results, if the result is positive for cancer, they will be invited to participate in intervention C. If the result is negative for cancer, they will be reassigned to intervention A, while remaining in their originally randomized group (experimental or control arm) (Fig. 1).

The randomization will be conducted using the Study Randomizer software (2017) [26] with a 1:1 allocation ratio and randomly varied block sizes of two and four. The allocation will be determined using permuted blocks [27], and the randomization process will be carried out by an independent researcher. Participants will not be blinded to their assigned intervention condition, which is common practice in psychological web-based interventions [28,29].

To ensure transparency in data analysis, all researchers will remain blinded to the participants' experimental conditions until all analyses have been completed.

### 2.3 Participants

The participants will be women attending the Breast Clinic of the Mexican Social Security Institute, a public hospital. These patients are referred from family medicine clinics for breast cancer screening. The clinic is one of ten in Mexico specifically dedicated to the prompt detection of breast cancer through screening and diagnostic studies.

#### 2.3.1 Selection criteria

Women will be included in the study if they meet the following criteria: 1) they are of legal age, 2) they attend the Breast Clinic for breast cancer screening and/or diagnostic evaluation, 3) they are capable of using an electronic device to access the clinic's virtual platform, and 4) they provide informed consent to participate in the study. Participants will be excluded if they have a cognitive disability that impairs their ability to navigate the platform or read the visual material.

#### 2.3.2 Procedure and data collection

Fig. 1 outlines the procedure and data collection process. After accepting the invitation, the participants will access the website www.saludmamaria.com, where they provide their cell phone number. They will then receive a link via WhatsApp, granting them access to each intervention.

The study variables will be measured using online questionnaires administered through the platform before and after each intervention (Table 1). For the active control group, an expert psychologist will administer the instruments, as these participants will not access the platform. Measurements will be taken both before and after each intervention. The duration of each intervention will vary depending on the patient, as each module will be made available only after the previous one is completed. However, based on the activities included in each module, the average time to complete each module is approximately one day. Therefore, it is estimated that post-assessment for the control group will take place after three days for interventions A and B, and after six days for intervention C.

**Table 1:** Study design.

	Enrollment	Allocation	Post-allocation								
		0	A			B			C		
			Pre-test	Intervention	Post-test	Pre-test	Intervention	Post-test	Pre-test	Intervention	Post-test
Inclusion											
Eligibility	X										
Informed consent	X										
Assignment		X									
**Interventions**											
Internet-Based Psychoeducational Program (experimental group)			X	X	X	X	X	X	X	X	X
Visual material (active control group)			X	X	X	X	X	X	X	X	X
**Assessments**											
Sociodemographic data			X			X			X		
***Primary outcomes***											
State-trait Anxiety Inventory			X		X	X		X	X		X
Psychological Consequences Questionnaire			X		X	X		X	X		X
***Secondary outcomes***											
Sense of Coherence Scale			X		X	X		X	X		X
***Other outcomes***											
Satisfaction with the Intervention					X			X			X
System Usability					X			X			X

### 2.4 Measures

#### 2.4.1 Primary outcomes

##### 2.4.1.1 State-trait anxiety Inventory - state subscale

The State-Trait Anxiety Inventory (STAI) is designed to assess both state anxiety (a temporary condition influenced by the current situation) and trait anxiety (a general tendency to experience anxiety) [30]. For this study, the state-anxiety subscale will be used. This subscale consists of 20 items that evaluate anxiety in the present moment. It has been validated in the Mexican adult population, demonstrating a Cronbach's alpha of 0.89 for the state-anxiety subscale [31].

##### 2.4.1.2 Psychological consequences questionnaire

The Psychological Consequences Scale (PCQ) assesses the negative psychological outcomes experienced as a result of thoughts or emotions related to breast cancer screening. It consists of 12 items with four Likert-type response options. The 12 items are divided into three subscales: 1) Emotional, 2) Physical, and 3) Social, with Cronbach's alpha values of 0.89, 0.77, and 0.78, respectively [32].

#### 2.4.2 Secondary outcomes

##### 2.4.2.1 Sense of Coherence Scale

The sense of Coherence Scale was originally developed by Antonovsky (1993) and later reformulated and validated in the Mexican university population by Velázquez-Jurado et al. [33], who reported a Cronbach's alpha of 0.792 for the overall scale. The scale comprises 13 items grouped into three dimensions: understandability, manageability, and meaningfulness. The total score ranges from 13 to 91 points, calculated by averaging across all dimensions.

#### 2.4.3 Other outcomes

##### 2.4.3.1 Satisfaction with the intervention

The Client Satisfaction Questionnaire (CSQ-8) [34] will be used to assess user satisfaction with the treatment. This scale consists of eight items that measure the level of satisfaction with the intervention. It has been utilized in previous evaluations of self-applied treatment and has demonstrated a high degree of internal consistency [35,36].

##### 2.4.3.2 System usability

The System Usability Scale (SUS) will be employed [37]. This scale consists of 10 items, rated on a 5-point scale, ranging from 0 (strongly disagree) to 4 (strongly agree), and is designed to evaluate the usability of a system.

### 2.5 Internet-Based Psychoeducational Program (IBPP) Description

The IBPP, “*Salud Mamaria*”, is a psychoeducational program delivered through an online platform (www.saludmamaria.com). It consists of three multicomponent interventions tailoreed for women who visit the Breast Clinic for breast cancer screening or diagnostic tests:

1. Intervention A – *Improving Your Health Habits and Self-Care*: This intervention is designed for women without suspicion of cancer or those who have recently completed their screening tests. It provides psychoeducational information focused on the prevention and early detection of breast cancer.
2. Intervention B – *Waiting for Your Biopsy Result*: Aimed at women who suspect they may have cancer and are awaiting biopsy results, this intervention provides emotional management strategies to support patients during the waiting period.
3. Intervention C – *Supporting You After Your Breast Cancer Diagnosis*: This intervention is offered to women who have received a positive cancer diagnosis. It provides psychoeducation information about breast cancer and its treatment, along with psychological strategies to support patients in the immediate days after following diagnosis.

The details of each intervention and their respective modules are outlined in Table 2.

**Table 2:** Description of the Internet-based psychoeducational program.

Intervention	Modules	Techniques
A: Improving Your Health Habits and Self-Care	Module 1: Self-examination and basic concepts about screening tests and diagnostic evaluation	Psychoeducation
		Self- monitoring
		Decisional balance
	Module 2: Prevention of risk factors associated with breast cancer	Psychoeducation
	Module 3: Diet and the association of Body Mass Index with cancer	Psychoeducation
		Self- monitoring
		Decisional balance
B: Waiting for the Result of Your Biopsy	Module 1: Fear about the biopsy result	Psychoeducation
	Module 2: Fear and anxiety. Components of anxiety (cognitive, behavioral, physiological)	Psychoeducation
		Self- monitoring
	Module 3: Breathing exercises to reduce anxiety	Psychoeducation
		Diaphragmatic breathing
C: Supporting You After Your Breast Cancer Diagnosis	Module 1: Medical aspects of breast cancer and treatment	Psychoeducation
	Module 2: Reflection on the treatment process and the gains and losses of cancer	Self-Reflective Emotional Writing
	Module 3: Coping strategies for treatment adherence	Problem-solving
	Module 4: Mindfulness	Mindfulness
		Self- monitoring
	Module 5: Social support	Communication techniques
	Module 6: Acceptance of the illness. Acceptance and commitment therapy	Values and committed action

**Fig. 2:**
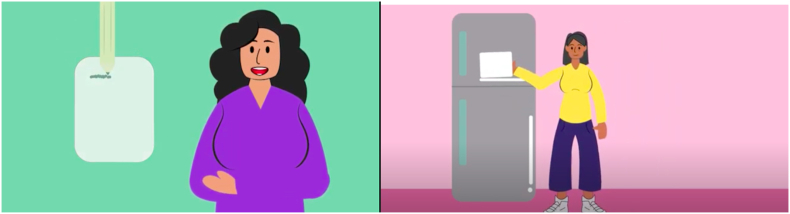
Animated videos for Intervention A.

Each module includes an animated video that explains the topic and demonstrates the associated techniques (Fig. 2).

The videos were produced by an interdisciplinary team that included Art and Interactive Technologies Designers as well as Graphic Designers. The videos were created in a motion graphics style, combining two-dimensional graphics with text, and occasionally incorporating real footage to demonstrate complex procedures. This combination of techniques was chosen to better engage the target audience and foster empathy through the characters’ design, use of color, and visual interactions.

#### 2.5.1 Structure of the IBPP platform

The interface of the IBPP platform was designed based on an analysis of social network interfaces, ensuring that the content is easily accessible and intuitive. A team of interactive designers developed the interface specifically for the target users (women around 50 years old with low levels of digital literacy) in an effort to reduce the drop-out rate.

To accommodate the needs of the patients and facilitate access, the procedure for inviting them to the platform is as follows:

1. Health personnel will invite patients to participate and assist them in registering their cell phone numbers on the website.
2. The patient will receive a WhatsApp message containing a link to the platform. They can leave and return to the website as often as they wish, with their progress being saved automatically.

**Fig. 3:**
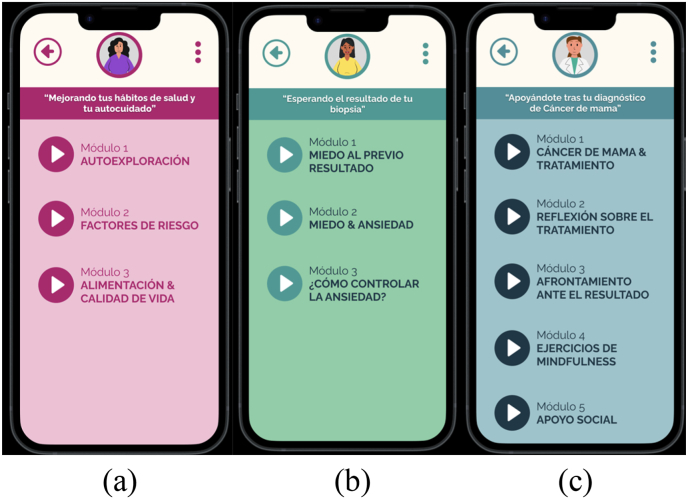
Prototype of the modules for the three interventions: a) Intervention A; b) Intervention B; c) Intervention C.

Upon entering the platform, participants will first encounter an introductory video designed to familiarize them with the platform's features. Following this, the initial questionnaires will be administered. The three interventions (A, B, or C) will then be presented, allowing participants to select the one that corresponds to their specific situation. After selecting an intervention, they will gain access to the relevant modules (Fig. 3).

##### 2.5.1.1 Active control group

**Fig. 4:**
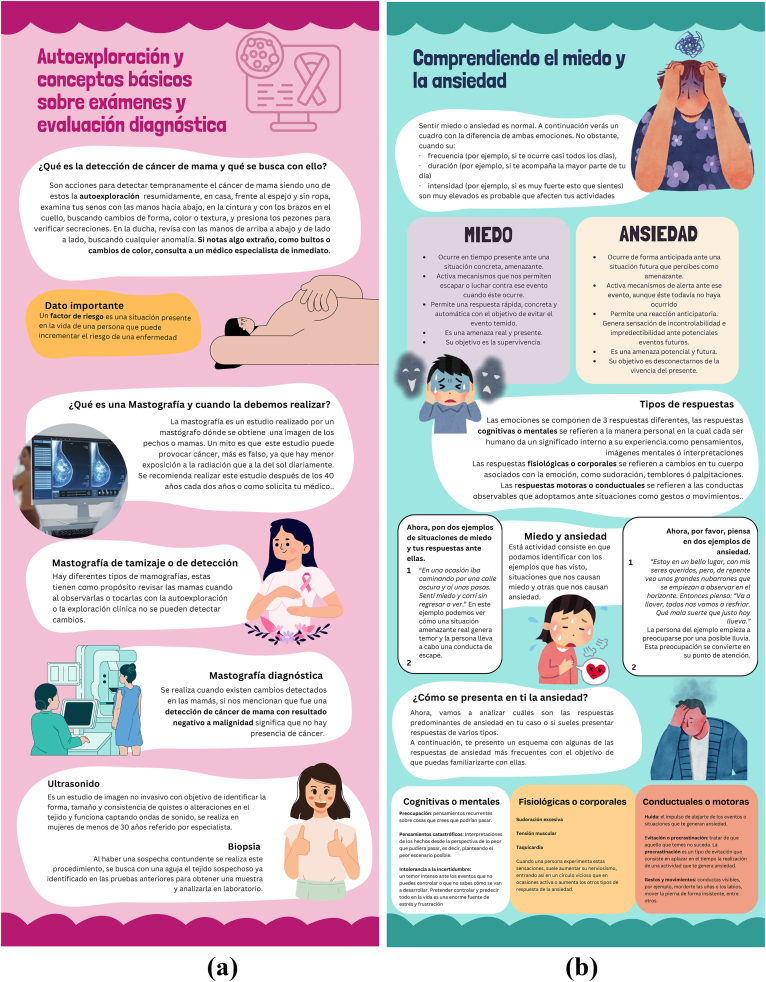
Examples of the psychoeducational materials for the active control group: a) Module 1 of Intervention A; b) Module 2 of Intervention B.

The active control group will receive printed psychoeducational materials corresponding to each intervention (A, B, and C). The topics covered will align with those presented on the platform (Table 2), but the content will consist solely of informational material without the inclusion of psychological techniques. Examples of the psychoeducational materials are shown in Fig. 4.

The interventions “A,” “B,” or “C” will be provided to patients at the breast clinic based on their specific medical condition (Fig. 1).

### 2.6 Potential risk for the participants

Adverse effects refer to potential negative events that participants may experience during the treatment, which are perceived as related to the intervention [38]. An email address will be made available to participants on the intervention's web platform to report any such events. Participants can contact the research team at any time to address concerns or report adverse effects related to or arising from the intervention. Upon receiving initial contact, a qualified clinical psychologist from the team will respond via email to explore the issue in detail and provide personalized solutions. Monitoring adverse effects effectively prevents them and helps reduce dropout rates in web-based interventions [38,39].

Additionally, to detect any worsening of target symptoms, scores for primary outcomes will be analyzed by comparing pre- and post-measurement for each participant. Participants showing a deterioration in symptoms will be contacted via email by a team member, who will offer support and provide information on external resources available to them.

### 2.7 Data analysis

A data monitoring committee (DMC) is not required for this study, as one of the authors (ADR) will have access to participant information, which is automatically saved on the platform, to monitor participation. The items from the STAI and PCQ scales will be summed according to their respective factors to generate scores for anxiety and negative psychological consequences associated with breast cancer. Similarly, the scores from the dimensions of the SOC scale will be averaged to produce an overall score for sense of coherence.

Four clinical variables will be measured pre-intervention for both the experimental and control groups, with the same variables measured post-intervention. Pre-intervention measurements for each group will be analyzed to assess the baseline similarity between groups. This analysis will also include the participants’ sociodemographic data. Numerical data will be reported as means and standard deviations, while categorical data will be presented as frequencies and percentages.

To evaluate the effect of the intervention, a linear model will be applied for each clinical variable, incorporating the effect of the time of measurement (pre-vs. post-intervention), the effect of the group (control vs. experimental), and the interaction between time and group. The interaction term will allow us to detect the intervention's effect over time (i.e., from pre-to post-treatment intervention). For anxiety and negative consequences variables, the interaction term is expected to be negative, indicating a reduction in scores for these clinical scales in the experimental group compared to the post-intervention control group. Conversely, the interaction term is expected to be positive for the sense of coherence, reflecting the beneficial impact of the treatment compared to the post-intervention control group.

The described model can address the question of the overall effectiveness of the intervention, regardless of whether participants received intervention A, B, or C. Since we cannot predict in advance how many participants will be included in each intervention, it was deemed inappropriate to propose a specific analysis for each type. However, considering that the women participating in each intervention may differ significantly from one another, and that a woman may participate in more than one intervention (e.g., moving from B to A or B to C), we will fit a second model. This model, in addition to the original specification, will include a categorical variable as a control predictor indicating the type of intervention received. The variable will have four levels:

1) Control – indicating women randomly assigned to the control condition;
2) A – indicating women without suspicion of cancer who directly received intervention A;
3) BA – indicating women with suspicion of cancer who received a negative biopsy result and who first received intervention B followed by A;
4) BC – indicating women with suspicion of cancer who received a positive biopsy result and who first received intervention B followed by C.

Both models will include additional control predictors such as age, diagnosis severity, and marital status. While this is not an exhaustive list of potential confounding variables, randomization will help control for their impact. After fitting the models, they will be compared based on R^2^, Akaike Information Criterion (AIC), and Bayesian Information Criterion (BIC) values, with the model showing the best fit indices being retained.

Subsequently post hoc differences between interventions will be explored by comparing post-treatment scores using a one-factor ANOVA. If F values associated with p < 0.05 are obtained, t-tests for independent samples will be conducted, with the false discovery rate (FDR) correction applied to adjust the p-value based on the number of comparisons performed. All analyses will be conducted using R, with a statistical significance level set at p < 0.05.

#### 2.7.1 Power analysis: sample size

To determine the sample size for this study, we used data from a randomized controlled trial that evaluated the effectiveness of an online self-help intervention for patients with glioma and depressive symptoms [40]. In that study, an effect size of Cohen's d = 0.65 was observed in the comparison between the treatment and control groups. Using the effect size, a Type I error rate of p = 0.05, and a power of 0.8, it was calculated that 60 participants (30 per group) are required to test a one-tailed hypothesis (e.g., the treatment group performing better than the control group). The sample size calculation was performed using the *pwr* package in R.

### 2.8 Ethical considerations

The study was approved by the Ethics Committee of the University of Guadalajara (approval number: CI-01022) on February 22, 2022. Additionally, the study is registered on ClinicalTrials.gov (ID: NCT05830461). Informed consent for the experimental group will be obtained through the platform, while a member of the research team will obtain consent for the active control group. Participation will be voluntary, and participants may decline to participate or withdraw their consent at any time without affecting their medical follow-up. All data collected will remain anonymous and confidential, with access restricted to a single researcher (ADR), who will be responsible for safeguarding the data. Privacy, confidentiality, and anonymity will be ensured, and the data will be used exclusively for research purposes in compliance with the Personal Data Protection Law.

## 3 Discussion

This article presents an Internet-Based Psychoeducational Program called “*Salud Mamaria*”, designed to provide psychoeducation to women undergoing the breast cancer diagnosis process. The program aims to reduce anxiety symptoms and negative psychological consequences while enhancing participants’ sense of coherence.

These online interventions are gaining popularity and acceptance in the health field as complementary components of comprehensive care [41,42]. Data from the well-being survey by the National Institute of Statistics and Geography in Mexico (INEGI) [43] reported that 80.8 % of respondents used social media, making the IBPP a viable and accessible tool for Mexican women to access information and strategies during their breast cancer diagnosis process. Additionally, the effectiveness of using WhatsApp for educational interventions targeting women undergoing screening studies has been demonstrated [44]. Our program aims to achieve this through three personalized interventions (A, B, and C).

Additionally, psychoeducation is an effective strategy for reducing distress in patients with chronic disease [45]. Lally et al. [46] highlight the importance of early distress management in cancer patients to enhance treatment adherence and improve psychological well-being. Conversely, they also noted that inadequate support for distress management in cancer patients is associated with non-adherence to treatment, reduced quality of life, and increased healthcare costs.

One of the strengths of the IBPP it that it was developed by an interdisciplinary team of experts, including physicians, psychologists, designers, and engineers, each contributing their expertise to the intervention's design. Before the development of the IBPP, interviews were conducted with patients who attended the Breast Clinic [20]. It was found that many patients were unaware of the screening tests they were about to undergo and had not sought information beforehand [20]. This feedback enabled the creation of a platform centered on patients' needs. Additionally, elements suggested by other studies to promote adherence to the app were incorporated, such as consideration of physical and sociocultural characteristics in the design of the avatars, ease of use, and an aesthetically pleasing interface [41].

Furthermore, the current protocol includes tailored interventions for patients without suspicion of cancer (intervention A) those with a suspicion of cancer awaiting biopsy results (intervention B), and follow-up for those who receive a positive cancer diagnosis (intervention C). This approach ensures that each group receives appropriate and relevant information based on their specific situation.

Finally, there are limited studies on the engagement and usability of eHealth tools, as well as the characteristics of the population using these apps in Latin America [41]. Understanding these barriers and identifying potential strengths will provide valuable insights for the effective implementation of such tools in healthcare.

One limitation of this study is the social equity barriers that may limit the use of smartphones and Internet among individuals with low resources or those living in extreme poverty in Mexico. According to data from INEGI [47], 84.1 million people in Mexico (66.4 % of the total population) have access to the Internet, and 80.96 million (63.9 %) have a smartphone. However, this disparity is more pronounced in rural areas, where only 50.4 % of the population has Internet access.

As a second limitation is that the application is currently available only in Spanish, restricting its use among indigenous communities or non-Spanish-speaking individuals. This issue could be addressed by verifying the application's effectiveness and expanding the collaboration network to other regions of Mexico and international communities.

## 4 Conclusion

This study presents a detailed protocol for delivering an Internet-Based Psychoeducational Program to women undergoing breast cancer screening or those with a confirmed diagnosis. “*Salud Mamaria*” has the potential to serve as an evidence-based tool for women in Mexico, a developing country where such resources are greatly needed but remain limited. This tool could be expanded and integrated into clinics across Mexico and other Latin American countries. Furthermore, implementing a psychoeducational program during the breast cancer screening and diagnosis process could promote the health and well-being of women while reducing misinformation and the distress associated with the process.

## CRediT authorship contribution statement

**Reyna Jazmín Martínez-Arriaga:** Writing – review & editing, Writing – original draft, Supervision, Project administration, Funding acquisition, Conceptualization. **Alejandro Dominguez-Rodriguez:** Writing – review & editing, Writing – original draft, Project administration, Investigation, Conceptualization. **Sergio Osvaldo Meza-Chavolla:** Writing – review & editing, Writing – original draft, Resources, Project administration, Funding acquisition. **Yineth Alejandra Muñoz-Anacona:** Writing – review & editing, Writing – original draft, Resources, Investigation, Conceptualization. **Adrián Antonio Cisneros-Hernández:** Writing – review & editing, Writing – original draft, Resources, Project administration, Conceptualization. **Joel Omar González-Cantero:** Writing – review & editing, Writing – original draft, Methodology, Investigation, Conceptualization. **Leivy Patricia González-Ramírez:** Writing – review & editing, Writing – original draft, Project administration, Investigation, Conceptualization. **Paulina Erika Herdoiza-Arroyo:** Writing – review & editing, Writing – original draft, Project administration, Methodology, Investigation. **Norma Alicia Ruvalcaba-Romero:** Writing – review & editing, Writing – original draft, Resources, Methodology, Investigation. **Fabiola Macías-Espinoza:** Writing – review & editing, Writing – original draft, Methodology, Investigation, Conceptualization. **Said Jiménez:** Writing – review & editing, Writing – original draft, Resources, Methodology, Investigation.

## Funding sources

This research did not receive any specific grant from funding agencies in the public, commercial, or not-for-profit sectors.

## Declaration of competing interest

The authors declare that they have no known competing financial interests or personal relationships that could have appeared to influence the work reported in this paper.

## Appendix A Supplementary data

Multimedia component 1 Standard Protocol Items: Recommendations for Interventional Trials (SPIRIT) statement.
